# Asthma Moderates the Association between the Big Five Personality Traits and Life Satisfaction

**DOI:** 10.3390/healthcare11182560

**Published:** 2023-09-16

**Authors:** Weixi Kang, Antonio Malvaso, Edward Whelan

**Affiliations:** 1 Department of Brain Sciences, Imperial College London, London W12 0BZ, UK; 2 Independent Researcher, 99MX QH Maynooth, Ireland; 3 Department of Brain and Behavioral Sciences, University of Pavia, 27100 Pavia, Italy

**Keywords:** asthma, life satisfaction, personality, Big Five

## Abstract

The current study aimed to examine whether asthma moderates the association between the Big Five personality traits and life satisfaction. By analyzing data from 3934 people with asthma (40.09% males) with a mean age of 49.2 (S.D. = 16.94) years old and 22,914 people without asthma (42.9% males) with a mean age of 45.62 (S.D. = 17.25) years old using a hierarchical regression and multiple regressions, the current study found that asthma significantly moderates the link between Neuroticism and life satisfaction and Openness and life satisfaction after controlling for other covariates. Specifically, Neuroticism was negatively related to life satisfaction whereas Agreeableness, Openness, Conscientiousness, and Extraversion were positively associated with life satisfaction in people with and without asthma. However, the negative association between Neuroticism and life satisfaction and the positive association between Openness and life satisfaction were stronger in people with asthma compared to people without asthma.

## 1. Introduction

Life satisfaction is a concept that measures how people appreciate or dislike their life [1]. In the literature, constructs such as subjective well-being, happiness, and life satisfaction are employed interchangeably [2]. This may not be strictly correct but there are undoubtedly similarities between the concepts [3]. Most people seek and are motivated by the need to acquire happiness, which can be difficult to define and measure in an objective way [3]. It is often claimed that subjective well-being can be relatively easily measured and identified [4]. Generally, there are two aspects of subjective well-being, and these are cognitive and affective well-being [5]. Affective well-being encompasses negative and positive emotional states and life satisfaction belongs to the cognitive aspect of subjective well-being [6].

It is necessary to understand the variables that contribute to life satisfaction, in particular for those patients with potentially life-limiting conditions such as asthma. This could help in the development of interventions that promote improved ratings of life satisfaction and promote improved physical, psychological, and behavioral aspects of a persona that is positively related to the quality of life. This can reduce the risk of mental health problems including depression while enhancing life expectancy [7], physical functioning [8,9], and social relationships [10,11].

There are a number of well-known theories on life satisfaction, such as top-down, bottom-down, and an integrated approach [1,2]. The top-down theory argues that life satisfaction is dependent on innate personality characteristics, produced by beneficial cognitive and affective states that prompt individuals to act in a stable manner [12]. Specific personality traits are held to be predictors of life satisfaction, which refers to factors that capture basic individual differences in terms of how one feels, thinks, and behaves. Among other personality models, the Big Five is one of the most widely used and can be translated into other models as well. Regarding the specific associations between the Big Five and life satisfaction, Neuroticism is believed to have a negative relationship with one’s perceived satisfaction with life. Traits including Agreeableness, Openness, Conscientiousness, and Extraversion are generally positively related to life satisfaction (e.g., [1,2,13]), although there are some inconsistencies [14,15,16,17,18,19,20,21]. On the other hand, the bottom-down approach regards life satisfaction as being a result of interacting variables such as satisfaction with health, economic status, social capital, leisure, and accommodation [1,2,12,22].

A recent trend in the research on the subject has seen an integrated model of life satisfaction become widely accepted by studies. This theory holds that positive reports of life satisfaction are dependent on a combination of demographic factors, aspects of life satisfaction, and personality characteristics. In general, life satisfaction remains consistent over many years [23,24,25], and this is aligned to the top-down theory that argues that personality traits are correlated with positive life satisfaction. This theory argues that life satisfaction can be altered and irrevocably changed by crises and adverse events such as major health issues or bereavement [26,27]. Moreover, dissatisfaction with one aspect of life can lead to a revaluation of another aspect of life. This can also lead to individuals re-examining their life, for example, a diagnosis of asthma can lead them to evaluate their work–life balance [1,2]. This indicates that it is possible to break the connection between life satisfaction and personality characteristics, as argued for in the integrated theory [1,2]. Moreover, the emotional and psychological toll of managing asthma might accentuate the influence of certain personality traits on an individual’s sense of contentment. Additionally, the constraints and adjustments necessitated by asthma could reshape how an individual’s inherent personality traits interact with their opportunities for social engagement and personal fulfillment, thereby shaping their overall life satisfaction in a nuanced manner. Specifically, individuals who are neurotic might experience heightened anxiety and distress due to their health condition, leading to a more intricate interplay between their personality traits and their overall sense of life satisfaction.

In general, the research indicates that asthma negatively impacts individuals’ quality of life. Studies have found a negative correlation between asthma and perceived life satisfaction [23,28,29]. As a result, it is possible to state with a high degree of confidence that asthma moderates the connection between personality and reports on life satisfaction. It is necessary to understand if a clinical diagnosis of asthma is a moderator in the relationship between personality traits and life satisfaction, given life satisfaction is closely related to a lot of outcomes such as morbidity and mortality. The aim of the current study is to test how asthma could moderate the association between personality traits and life satisfaction. We hypothesize that asthma may moderate the association between Neuroticism and life satisfaction.

## 2. Methods

### 2.1. Data

The research utilized data from the UK Household Longitudinal Study (UKHLS), known as the Understanding Society [30], which has been gathering annual information from a group of UK households since 1991. Ethical committees at the University of Essex have approved all data collections, and participants provided informed consent before the study began. Initially, participants were asked in Wave 1 (collected between 2009 and 2010) whether they had received a clinical diagnosis of asthma. Subsequently, in each wave up until Wave 3, participants were asked if they had been newly diagnosed with asthma. Furthermore, at Wave 3 (collected between 2011 and 2012), participants completed questionnaires addressing personality traits, demographic information, and psychological distress. The data from participants who could not provide information on all the variables sought were not included in the analysis. Thus, there were 3934 people with asthma (40.09% males), with a mean age of 49.2 (S.D. = 16.94) years old, and 22,914 people without asthma (42.9% males), with a mean age of 45.62 (S.D. = 17.25) years old.

### 2.2. Measures

#### 2.2.1. Asthma

Participants answered the question “Has a doctor or other health professional ever told you that you have any of these conditions? Asthma.” to indicate if they have asthma. Self-reported asthma has been proven to be a valid measure of asthma status in various countries [31,32,33,34,35,36,37]. This question has been asked in Wave 1 to Wave 3. Participants who indicated that they were diagnosed with asthma between Wave 1 to Wave 3 and were coded as people with asthma.

#### 2.2.2. Personality Traits

To assess personality traits, the study employed the 15-item version of the Big Five Inventory [38], where participants rated their agreement on a Likert scale ranging from 1 (“disagree strongly”) to 5 (“agree strongly”), which captures the personality traits Neuroticism, Agreeableness, Openness, Conscientiousness, and Extraversion. In cases where necessary, scores were reversed. The specific questions posed to participants can be accessed at https://www.understandingsociety.ac.uk/documentation/mainstage/dataset-documentation/term/personality-traits?search_api_views_fulltext=, (accessed on 1 March 2023). 

#### 2.2.3. Life Satisfaction

Participants were asked to rate their satisfaction with their overall life using a 7-point scale, ranging from 1 (not satisfied at all) to 7 (completely satisfied). It has been observed that the outcomes obtained from both single-item measures, such as the question mentioned, and multi-item measures like the Satisfaction with Life Scale (SWLS), yield comparable results [39].

#### 2.2.4. Control Variables

The demographic variables consisted of age, gender, monthly income, the highest level of education completed, marital status, and psychological distress, which was assessed using the GHQ-12 [40], which is a tool that measures mental health (alpha = 0.90). The specific codings for these variables are available in Table 1.

### 2.3. Analysis

To examine the potential moderating role of asthma in the relationship between personality traits and life satisfaction, a hierarchical regression analysis was employed. This analysis included asthma status, personality traits, demographic variables such as age, sex, monthly income, highest educational qualification, marital status, and the number of close friends, as well as the interaction between asthma status and personality traits to predict life satisfaction. As a post hoc analysis, two separate multiple regression models were conducted to predict life satisfaction in individuals with and without asthma. These models considered demographic variables, including age, sex, monthly income, highest educational qualification, marital status, psychological distress, and personality traits as predictors. All statistical analyses were carried out using MATLAB 2018a; *p*-values less than 0.05 were considered significant.

## 3. Results

**Figure 1:**
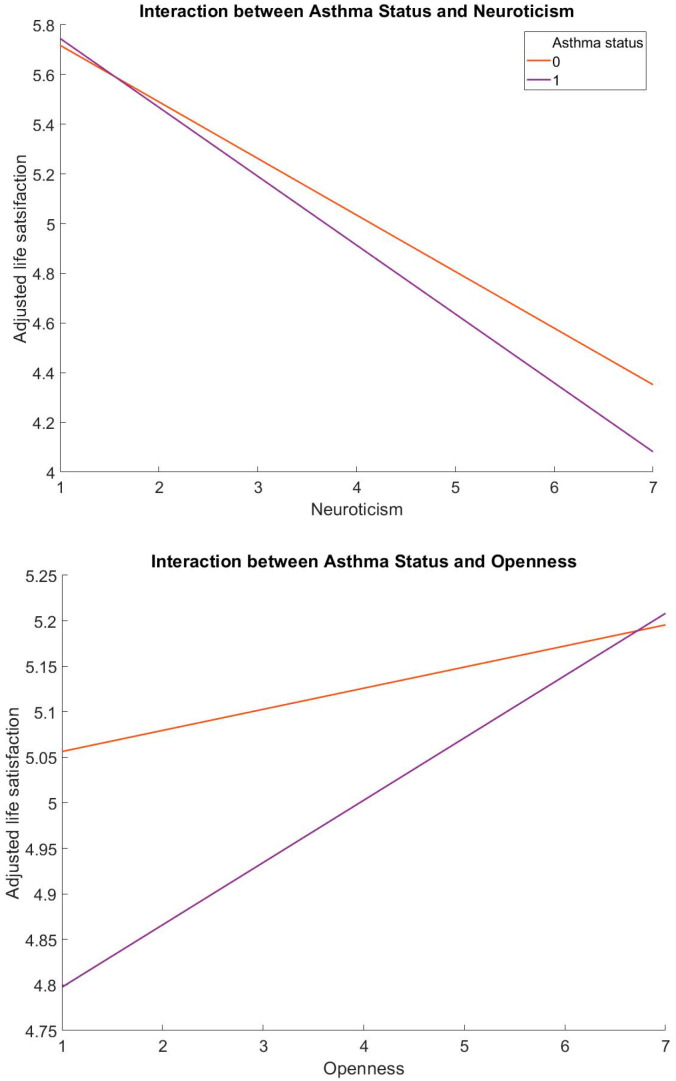
The moderating effect of asthma status in the association between Neuroticism, Openness, and life satisfaction.

The current study found that asthma significantly moderates the association between Neuroticism (*b* = −0.05, *p* < 0.01, 95% C.I. [−0.08, −0.02]) and life satisfaction, and Openness (*b* = 0.05, *p* < 0.05, 95% C.I. [0.01, 0.08]) and life satisfaction (Table 2, Figure 1). Specifically, Neuroticism was negatively related to life satisfaction (*b* = −0.23, *p* < 0.001, 95% C.I. [−0.24, −0.21]) whereas Agreeableness (*b* = 0.06, *p* < 0.001, 95% C.I. [0.04, 0.08]), Openness (*b* = 0.03, *p* < 0.01, 95% C.I. [0.01, 0.04]), Conscientiousness (*b* = 0.11, *p* < 0.001, 95% C.I. [0.09, 0.13]), and Extraversion (*b* = 0.08, *p* < 0.001, 95% C.I. [0.07, 0.10]) were positively related to life satisfaction in people without asthma. Similarly, Neuroticism was negatively related to life satisfaction (*b* = −0.28, *p* < 0.001, 95% C.I. [−0.31, −0.25]) whereas Agreeableness (*b* = 0.05, *p* < 0.05, 95% C.I. [0.00, 0.09]), Openness (*b* = 0.05, *p* < 0.01, 95% C.I. [0.02, 0.09]), Conscientiousness (*b* = 0.09, *p* < 0.001, 95% C.I. [0.04, 0.13]), and Extraversion (*b* = 0.11, *p* < 0.001, 95% C.I. [0.07, 0.14]) were positively related to life satisfaction in people with asthma (Table 3).

## 4. Discussion

The aim of the current study was to test whether asthma moderates the association between personality traits and life satisfaction. Results showed that asthma significantly moderated the link between Neuroticism and life satisfaction and Openness and life satisfaction after controlling for other covariates. Specifically, Neuroticism was negatively related to life satisfaction whereas Agreeableness, Openness, Conscientiousness, and Extraversion were positively associated with life satisfaction in people with and without asthma. However, the negative association between Neuroticism and life satisfaction and the positive association between Openness and life satisfaction were stronger in people with asthma compared to people without asthma.

A negative relationship was found to exist between Neuroticism and life satisfaction, and this was expected based on previous research (e.g., [1,2,13]). Those individuals who have an elevated level of Neuroticism usually are less emotionally stable and are likely to have more negative emotions and they in general are more likely to experience difficulties in adverse situations. A link was found between Neuroticism and predictors of health such as walking speed and levels of exercise and a number of physical and mental dysfunctions (e.g., [41,42]). Neuroticism is also related to serious depression [43] and Alzheimer’s disease [44]. Moreover, the worldview and outlook of those with Neuroticism influence in a highly negative way they examine and interpret their life experiences, and this in turn leads to low levels of reported satisfaction with their life. Moreover, the negative association between Neuroticism and life satisfaction was stronger in asthma patients compared to the control population, which did not have asthma. This can be partly explained by the fact that Neuroticism is a predictor of several serious mental health conditions including anxiety and major depression (e.g., [45,46,47,48,49]). It is also well known that asthma patients are more prone to worse mental health, which can be very debilitating in comparison to non-asthmatic individuals. In addition, neurotic people with asthma may be more worried about their health condition, which can lead to a less satisfied life. In addition, individuals with asthma were characterized by higher Neuroticism scores, which may explain why their Neuroticism score has a stronger negative association with life satisfaction compared to controls.

Generally, those who have a high rating in Openness are more willing to have new experiences. They are always seeking something new and dislike routine. As a result, they are often drawn into interests that are stimulating and willing to take part in activities that promote their personal growth and development (e.g., [50]). This, in turn, means that those who rate their Openness as high are pursuing interests that satisfy their social and psychological needs and this generally translates into positive assessments of their life satisfaction. Those who have the characteristic of Openness are more likely to engage in healthy lifestyles (e.g., [41,42,51,52]). In addition, the association between Openness and life satisfaction was stronger in people with asthma, which can be explained by the fact that Openness is characterized by a willingness to adapt to change and embrace uncertainty. Individuals with asthma may need to navigate unexpected health fluctuations and adapt their daily routines to accommodate their condition. Openness might enable them to view these adaptations as opportunities for growth rather than as limitations, leading to increased life satisfaction. In addition, Openness is linked to engaging in creative and intellectually stimulating activities. People with asthma might channel their openness into hobbies, artistic pursuits, or other activities that bring them joy and a sense of accomplishment. These activities can contribute significantly to life satisfaction, especially in the face of health challenges.

Those individuals who rate their Extraversion as high are confident and outgoing. Extroverts are more positive and optimistic about the world, and this leads to positive perceptions about their quality of life. One of the characteristics of those who score high on Extraversion is that they are more physically active [52,53], giving them quality sleep [41], and are less likely to be depressed and stressed [43], which leads to higher levels of life satisfaction. Asthma patients may have only limited work opportunities and limited finances may result in them being less socially active. This may explain why asthma patients who generally have low Extraversion are more likely to report low life satisfaction.

Conscientiousness is a trait that is associated with being task-focused and orderly. This research has found that this trait has a positive association with life satisfaction in asthma sufferers and the healthy control population, as in previous research findings (e.g., [1,2,13]). Those individuals with prominent levels of Conscientiousness usually seek to place themselves in contexts that meet their goals and needs [16,54,55]. They are then more likely to achieve goals and satisfy needs and this gives them a sense of achievement, and this produces higher ratings of life satisfaction [16,56].

The strength of the current study includes well-controlled demographics and a healthy control population. Nevertheless, there are some limitations as in any study. The research is based on self-reported data without clinical assessment of severity, and this unavoidably results in some self-reporting biases. In the future, research on the subject should utilize more objective measurements, for example, clinical examinations. This study is a cross-sectional one, and this limits any conclusions about causation. Further research should adopt a longitudinal approach to determine any or all causal relationships. Another limitation of this study is that it did not measure specific types of asthma. It is necessary to understand if a given personality or trait has a positive relationship to one of the categories of asthma.

## 5. Conclusions

Taken together, the current study found that asthma moderates the association between Neuroticism and life satisfaction and Openness and life satisfaction. Health professionals should use the findings to support the development of prevention strategies and interventions such as reducing Neuroticism and increasing Openness to improve life satisfaction in people with asthma. Doing so could help asthma patients achieve the best outcomes in the physical, psychological, and behavioral domains.

## Figures and Tables

**Table 1:** Descriptive statistics for people with and without asthma.

	Healthy Controls		People with Asthma	
	Mean	S.D.	Mean	S.D.
Age	49.20	16.94	45.62	17.25
Monthly income	1446.76	1409.08	1359.30	1107.52
The number of close friends	5.21	5.35	5.09	4.58
Life satisfaction	5.15	1.50	4.96	1.56
Neuroticism	3.51	1.46	3.76	1.49
Agreeableness	5.66	1.04	5.65	1.06
Openness	4.53	1.34	4.60	1.33
Conscientiousness	5.51	1.10	5.44	1.11
Extraversion	4.57	1.35	4.64	1.32
	N	%	N	%
Sex				
Male	9831	42.90	1577	40.09
Female	13,083	57.10	2357	59.91
Highest educational qualification				
Below college	15,642	68.26	2623	66.68
College	7272	31.74	1311	33.32
Legal marital status				
Single	10,054	43.88	2031	51.63
Married	12,860	56.12	1903	48.37

**Table 2:** The estimates (*b*) of multiple regression models with the interactions of personality traits and asthma status.

	*b*
Age	0.00 ***
Sex	0.13 ***
Monthly income	0.00 ***
Highest educational qualification	0.10 ***
Legal marital status	0.25 ***
Psychological distress	0.01 ***
Neuroticism	−0.23 ***
Agreeableness	0.06 ***
Openness	0.02 **
Conscientiousness	0.11 ***
Extraversion	0.08 ***
Asthma status	0.05
Asthma status × Neuroticism	−0.05 *
Asthma status × Agreeableness	−0.02
Asthma status × Openness	0.05 *
Asthma status × Conscientiousness	−0.03
Asthma status × Extraversion	0.03
R^2^	0.11

* *p* < 0.05, ** *p* < 0.01, *** *p* < 0.001

**Table 3:** The estimates (*b*) of multiple regression models for healthy controls and people who have been diagnosed with asthma by taking demographics, the number of close friends, and personality traits as the predictors and life satisfaction as the predicted variable. All numbers are rounded up to two digits.

	Healthy Controls	People with Asthma
Age	0.00 ***	0.00
Sex	0.14 ***	0.06
Monthly income	0.00 ***	0.00
Highest educational qualification	0.10 ***	0.13 *
Legal marital status	0.25 ***	0.22 ***
Psychological distress	0.01 ***	0.01 *
Neuroticism	−0.23 ***	−0.28 ***
Agreeableness	0.06 ***	0.05 *
Openness	0.03 ***	0.05 **
Conscientiousness	0.11 ***	0.09 ***
Extraversion	0.08 ***	0.11 ***
R^2^	0.105	0.129

* *p* < 0.05, ** *p* < 0.01, *** *p* < 0.001

## Data Availability

Data can be found at https://www.understandingsociety.ac.uk/documentation/mainstage, (accessed on 1 March 2023).
